# Effects of Simulated Heat Waves on ApoE-/- Mice

**DOI:** 10.3390/ijerph110201549

**Published:** 2014-01-28

**Authors:** Chunling Wang, Shuyu Zhang, Ying Tian, Baojian Wang, Shuanghe Shen

**Affiliations:** 1 School of Applied Meteorology, Nanjing University of Information Science and Technology, 219 Ningliu Road, Nanjing 210044, China; E-Mails: wangchunling668@126.com (C.W.); yngtian@163.com (Y.T.); yqzhr@nuist.edu.cn (S.S.); 2 Key Laboratory of Arid Climatic Change and Reducing Disaster of Gansu Province, Lanzhou Institute of Arid Meteorology, China Meteorological Administration, 2070 Donggang East Road, Lanzhou 730020, China; 3 Lanzhou Central Meteorological Observatory, 2070 Donggang East Road, Lanzhou 730020, China; E-Mail: baojianwang@126.com

**Keywords:** heat wave weather, SOD, ApoE-/- mice, coronary disease, BH4

## Abstract

The effects of simulated heat waves on body weight, body temperature, and biomarkers of cardiac function in ApoE-/- mice were investigated. Heat waves were simulated in a meteorological environment simulation chamber according to data from a heat wave that occurred in July 2001 in Nanjing, China. Eighteen ApoE-/- mice were divided into control group, heat wave group, and heat wave BH4 group. Mice in the heat wave and BH4 groups were exposed to simulated heat waves in the simulation chamber. Mice in BH4 group were treated with gastric lavage with BH4 2 h prior to heat wave exposure. Results showed that the heat waves did not significantly affect body weight or ET-1 levels. However, mice in the heat wave group had significantly higher rectal temperature and NO level and lower SOD activity compared with mice in the control group (*p* < 0.01), indicating that heat wave had negative effects on cardiac function in ApoE-/- mice. Gastric lavage with BH4 prior to heat wave exposure significantly reduced heat wave-induced increases in rectal temperature and decreases in SOD activity. Additionally, pretreatment with BH4 further increased NO level in plasma. Collectively, these beneficial effects demonstrate that BH4 may potentially mitigate the risk of coronary heart disease in mice under heat wave exposure. These results may be useful when studying the effects of heat waves on humans.

## 1. Introduction

Heat waves, as a type of extreme weather, have significant impacts on human health [1]. Duration and frequent occurrence of high-temperature weather leads to increased hospitalization and mortality of patients with cardiovascular disease. The number of deaths from cardiac events caused by hot weather in China has increased and contributed to the increasing prevalence of coronary heart disease in China, which reached 6.49% in 2004 [2]. Nanjing, a big city in Eastern China with the nickname “stove”, is well known for its high summer temperatures. In recent years, the average summer temperature in Nanjing has shown a significant upward trend, with more frequent occurrence of heat waves than the national average, likely attributable to global warming [3]. Consequently, the rate of heart diseases such as coronary disease has significantly increased in Nanjing over the years [4,5]. In the present study, we aimed to investigate effects of heat waves on cardiac function in ApoE-/- mice, a well-established animal model for atherosclerosis [6,7,8]. ApoE-/- mice were placed in a meteorological environment simulation chamber and exposed to simulated heat waves according to heat wave data recorded in Nanjing. Cardiac functions of ApoE-/- mice were monitored by levels of cardiac biomarkers, including body temperature and levels of Superoxide Dismutase (SOD), Endothelin-1 (ET-1) and Nitric Oxide (NO). SOD levels start to change in the early stages of cardiovascular disease and thus are a sensitive biomarker for the diagnosis of coronary heart disease and other closely related diseases. Moreover, we studied effects of exogenous tetrahydrobiopterin (BH4) on cardiac functions of ApoE-/- mice exposed to simulated heat waves. BH4 is an important cofactor of NO synthase (NOS). Research shows that NO content decreases with decreasing level of BH4, resulting in impaired vascular expansion and poor body heat dissipation. Additionally, studies have shown that exogenous BH4 significantly prevents endothelial cell dysfunction, increases NO levels, promotes angiectasis recovery, increases heat dissipation, and helps body adaptation to heat. Therefore low levels of BH4 are believed to play a role in cerebrovascular diseases, especially in patients exposed to high temperatures and heat waves [9]. Finally, we discussed the potential effects of heat waves on coronary heart diseases in humans based on our experimental results obtained in ApoE-/- mice.

## 2. Experimental Section

### 2.1. Meteorological Environmental Simulation Chamber

The TEM1880 meteorological environment simulation chamber was provided by Tianjin Sprint Environmental Test Equipment Co., Ltd. (Tianjin, China). The chamber is capable of simulating a combined temperature-humidity-pressure test environment in the temperature range from −30 °C to 120 °C with ±0.5 °C fluctuations, humidity range of 30% to 98% with ±3% Relative Humidity (RH) fluctuations when the humidity is higher than 75% RH or ±5% RH fluctuations when the humidity is lower than 75% RH. The chamber also allowed fresh air (oxygen) injection when necessary during the experiments. A TH212 special thermal detector from Hong’ou Science and Technology Co., Ltd. (Beijing, China) was used in the study. The thermal detector had a measurable temperature range of −30 °C to 50 °C with accuracy of ±0.2 °C and resolution of 0.1 °C. A balance and an enzyme-linked immunity analyzer from G&G Measurement Plant (Changshu, China) were also used.

### 2.2. Modeling and Grouping of Animals

Eighteen 8-week-old Specific Pathogen Free (SPF) male ApoE-/- mice with average weight of 27.46 g were fed with high-fat diet for 8 weeks in plastic and metal cages under a circadian rhythm of 12 h/12 h with light supply from 08:00 to 20:00. The cages were kept at 27 °C (average summer temperature in Nanjing) with relative humidity of 45%. Noise in the breeding room was kept below 60 dB (A). The high-fat diet containing 10% lard, 10% cholesterol, 2% cholate, and 78% basal feed was purchased from Beijing Ke’ao Xieli Feed Co., Ltd. (Beijing, China). The high fat diet has been proved to induce atherosclerosis in rodents [6]. ApoE-/- mice fed for 8 weeks on this diet develop visible AS plates and thickening in aorta and coronary artery wall.

The eighteen ApoE-/- mice were randomly divided into three groups including control group (n = 6), heat wave group (n = 6), and heat wave BH4 group (n = 6). After the control period, mice in the heat wave and heat wave BH4 groups were exposed to a complete heat wave treatment. Heat wave BH4 group received gastric lavage with 10 mg/kg BH4 2 h prior to the heat wave treatment. Control and heat wave groups received gastric lavage with equal amounts of standard saline. Mice in the control group were kept in the same environment except for the heat wave treatment. All ApoE-/- mice were purchased from Vital River Laboratories (Beijing, China).

**Figure 1:**
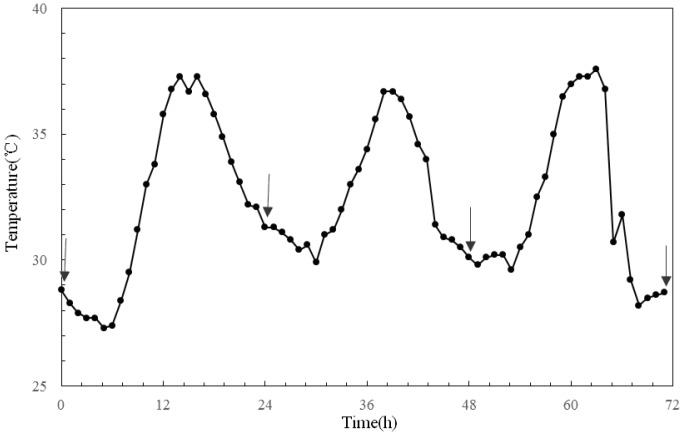
Temperature curve of simulated heat wave. The *arrows* stand for sampling time points when body temperature and body weight of ApoE-/- mice were measured in the three experimental groups.

### 2.3. Establishment of a Heat Wave Model

According to the China Meteorological Administration criteria, a maximum temperature ≥ 35 °C on a given day is called high temperature, and three or more consecutive days of high-temperature is called a heat wave. We developed a heat wave model based on hourly meteorological data (temperature, humidity, and precipitation) collected in Nanjing during the summer seasons (June to August) from 2001 to 2010. A heat wave occurred in July 2001 in Nanjing was used to establish the temperature simulation curve (Figure 1) [10,11]. The experimental temperature of the control group was set at 27 °C, the average summer temperature in Nanjing during 2001–2010.

### 2.4. Experimental Scheme

After grouping, all ApoE-/- mice were kept under the same feeding and environmental conditions as those in the adaptation period for 1 week. Heat wave and heat wave BH4 groups were exposed to heat wave treatment as shown in Figure 1 in the meteorological environment simulation chamber. Body weight and temperature of ApoE-/- mice in each group were measured before, during, and after the treatment at specific time points illustrated in Figure 1. After 3 days of heat wave exposure, ApoE-/- mice were removed from the chamber, anaesthetized (7% chloral hydrate, 0.3 mL/100 g), and blood samples (approximately 1 mL each) were collected by decollation into centrifuge tubes. Plasma samples were separated 15 min later and stored in refrigerators at low temperature (−80·°C) until analysis. ET-1 level in plasma was measured using an ELISA assay kit from EIAAB Science Co., Ltd. (Wuhan, China). Plasma NO level was determined by the nitrate reductase method using a reagent from Biological Engineering Research Institute of Nanjing (Nanjing, China). SOD level in cardiac tissues was determined as follows: The heart was extracted and the heart apex was weighed. Heart tissue was homogenized in 0.9% saline and centrifuged at 3,000 rpm for 15 min. The supernatant was collected and SOD concentration in redissolved cardiac tissue fluid was measured using an SOD measurement kit from the Biological Engineering Research Institute of Nanjing.

### 2.5. Statistical Analysis

Data were analyzed with SPSS19.0 and shown as mean ± SE (standard error). Differences between treatment groups were interpreted by one-way ANOVA with the LSD (least square difference) post hoc multiple comparisons. Differences with *p* < 0.05 were considered statistically significant.

## 3. Results and Discussion

### 3.1. Results

#### 3.1.1. Body Weight and Rectal Temperature

**Figure 2:**
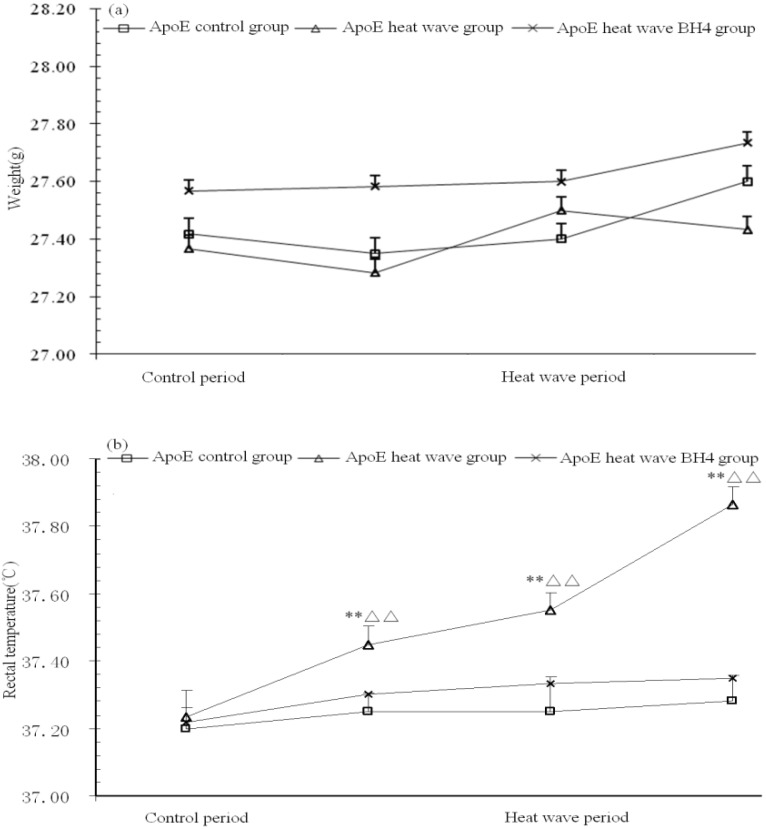
Body weight (**a**) and rectal temperature (**b**) of ApoE-/- mice. ** *p* < 0.01 *vs.* control group; △△ *p* < 0.01 *vs.* heat wave BH4 group at time of measurement.

Body weight and rectal temperature of ApoE-/- mice were monitored daily. As shown in Figure 2, body weight had increased slightly in all three groups by the end of the experiment (*p* > 0.05). Rectal temperature increased obviously by 0.7 °C in the heat wave group, while the heat wave BH4 and control groups showed only slight increases of 0.2 °C and 0.1 °C, respectively, in rectal temperature. Rectal temperature increases in the heat wave group were significantly higher than those in control group (*p* < 0.01); however, the rectal temperature differences between heat wave BH4 group and control group were statistically insignificant (*p* > 0.05).

#### 3.1.2. SOD Activity

**Figure 3:**
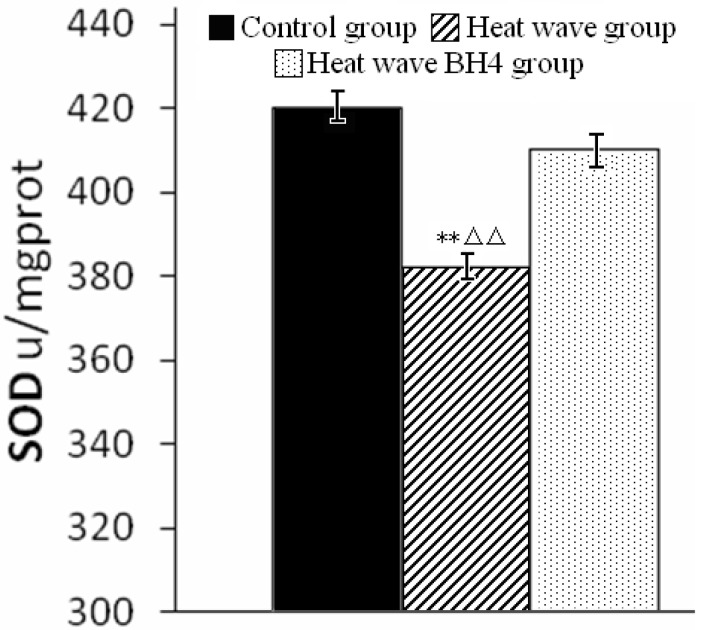
SOD activity in ApoE-/- mice at the end of the experiment. ** *p* < 0.01 *vs.* control group; △△ *p* < 0.01 *vs.* heat wave BH4 group.

At the end of the experiment, SOD activity in the heat wave group was significantly lower than in control group (*p* < 0.01). However, SOD activity in the heat wave BH4 group, although slightly lower than control (*p* > 0.05), was significantly higher than that in the heat wave group (*p* < 0.01) (Figure 3). 

Our results showed that three days of exposure to high temperatures resulted in a decrease in SOD activity in ApoE-/- mice, while BH4 treatment mitigated the heat wave-induced reduction in SOD activity.

#### 3.1.3. Levels of ET-1, NO, and ET-1/NO

At the end of the experiment, ApoE-/- mice in the three groups showed similar plasma ET-1 levels (Table 1). P-values associated with ANOVA for the three groups were greater than 0.05. Table 1 also shows NO levels in ApoE-/- mice at the end of the experiment. NO levels in the heat wave and heat wave BH4 groups were both significantly higher than those in control group (*p* < 0.01). Furthermore, the NO level in the heat wave BH4 group was significantly higher than that in the heat wave group (*p* < 0.01). NO/ET-1 ratios in ApoE-/- mice at the end of the experiment are also shown in Table 1. Since ET-1 levels were similar in the three groups, differences in NO/ET-1 ratios largely resembled those in NO levels. While the NO/ET-1 ratio in the heat wave group was slightly significantly higher than that in control group (*p* < 0.05), the NO/ET-1 ratio in the heat wave BH4 group was significantly higher than that in control group (*p* < 0.01). Furthermore, the NO/ET-1 ratio in the heat wave BH4 group was significantly higher than that in the heat wave group (*p* < 0.01). 

**Table 1:** ET-1 level, NO level and NO/ET-1 ratio in ApoE-/- mice at the end of the experiments.

		Group(s)	ET-1 (ng/L)	NO (μmol/L)	NO/ET-1 (%)
**Mean ± SE**		Control	167.84 ± 8.59	52.17 ± 5.92	31.13 ± 3.57
		Heat wave	165.82 ± 8.77	62.53 ± 5.07	37.84 ± 4.22
		Heat wave BH4	166.93 ± 12.98	96.99 ± 6.45	58.32 ± 5.05
***P* value**	**of ANOVA among**	the all three groups	0.945	0.000	0.000
	**of post hoc test between**	Control & heat wave	-	0.008	0.017
		Control & heat wave BH4	-	0.000	0.000
		Heat wave & heat wave BH4	-	0.000	0.000

### 3.2. Discussion

Pathological features of atherosclerosis in ApoE-/- mice are close to those of atherosclerosis in humans. Therefore we chose to use a ApoE-/- mouse model to study the effects of heat waves on coronary heart disease. NO and ET-1 are important regulators of cardiovascular functions. They have vital roles in maintaining basal vascular tone and cardiovascular homeostasis [12], and changes in their levels affect diastole and contraction of blood vessels. Vascular relaxation directly affects body heat loss. Heat wave-induced angiectasis promotes body heat loss through body surface, which helps prevent heat-induced body damage. Therefore, NO and ET-1, as key regulators of vascular relaxation, are biomarkers of cardiac function under heat wave exposure. SOD is an antioxidant enzyme that clears peroxide radicals generated from lipid peroxidation [13,14] and thereby protects the organism from free radical-induced damage. Studies have reported that SOD levels decrease with high temperature exposure in animals [15]. Therefore, SOD level can be at least partly or indirectly a bio-marker for cardiac function.

In this study, we monitored body weight, rectal temperature, levels of plasma ET-1 and NO, and heart SOD activity in ApoE-/- mice exposed to three consecutive days of simulated heat wave. Our results showed that heat wave exposure did not have a significant effect on body weight. While rectal temperature increased significantly in the heat wave group, gastric lavage with BH4 significantly reduced heat wave-induced rectal temperature increases (Figure 2b). Our results also showed that although ET-1 levels in the three groups of ApoE-/- mice showed no significant differences after heat wave exposure, the NO level in the heat wave group was 10.36 μmol/L higher than that in the control group, implying that heat wave exposure increased vasodilation and heat dissipation in ApoE-/- mice, as a defense mechanism against heat-induced increase in body temperature. Importantly, gastric lavage with BH4 in the BH4 group further increased NO levels compared with the heat wave group (34.46 μmol/L, *p* < 0.01), suggesting that BH4 treatment further promoted vascular dilation and heat dissipation in ApoE-/- mice during heat wave exposure. Collectively, our results suggested that BH4 promotes cardiac function in ApoE-/- mice during heat wave treatment and thus may potentially help reduce the incidence of heat wave-induced coronary heart disease in mice. 

Our results also showed that heat wave exposure significant decreased SOD activity in ApoE-/- mice. Gastric lavage with BH4 mitigated heat waved-induced decrease in SOD activity, indicating that BH4 may protect ApoE-/- mice from heat-induced cardiac damage via maintaining SOD activity. These results may be useful when studying the effects of heat waves on cardiac function in humans.

## 4. Conclusions

In summary, heat wave exposure significantly increased body temperature and NO level and decreased SOD activity in ApoE-/- mice, while having little effect on ET-1 levels. BH4 mitigated heat wave-induced increases in body temperature and decreases in SOD activity. Additionally, BH4 further elevated NO levels in ApoE-/- mice under heat wave exposure. Our results indicate that BH4 may protect ApoE-/- mice from heat-induced cardiac damage by promoting vascular relaxation and heat dissipation and maintaining efficient clearance of harmful free radicals. Collectively, these beneficial effects demonstrate that BH4 may potentially mitigate the risk of coronary heart disease in mice under heat wave exposure. Further studies are needed to assess the role of these results on cardiac function and coronary heart disease in humans under heat wave exposure.
